# Bis(4-pyrid­yl) disulfide–2,2′-[(*p*-phenyl­enebis(­oxy)]diacetic acid (1/1)

**DOI:** 10.1107/S1600536811025694

**Published:** 2011-07-06

**Authors:** Guang-Yin Wang

**Affiliations:** a Department of Chemistry, Dezhou University, Dezhou, Shandong 253023, People’s Republic of China

## Abstract

The asymmetric unit of the title 1:1 co-crystal, C_10_H_8_N_2_S_2_·C_10_H_10_O_6_, comprises two half-mol­ecules, the bis­(4-pyrid­yl) disulfide having twofold rotational symmetry and the 2,2′-[(*p*-phenyl­enebis(­oxy)]diacetic acid having crystallographic inversion symmetry. In the disulfide mol­ecule, the dihedral angle between the two pyridine rings is 86.8 (1)°, while the carboxyl groups of the substituted quinone lie essentially in the plane of the benzene ring [dihedral angle = 5.3 (1)°]. In the crystal, the components are linked *via* inter­molecular O—H⋯N hydrogen bonds into zigzag chains which extend along *c* and are inter­linked through C—H⋯π associations.

## Related literature

For the use of bis­(4-pyrid­yl)disulfide (bpds) as a linker in the construction of coordination polymers, see: Kondo *et al.* (2000 ▶); Zhu *et al.* (2010 ▶).
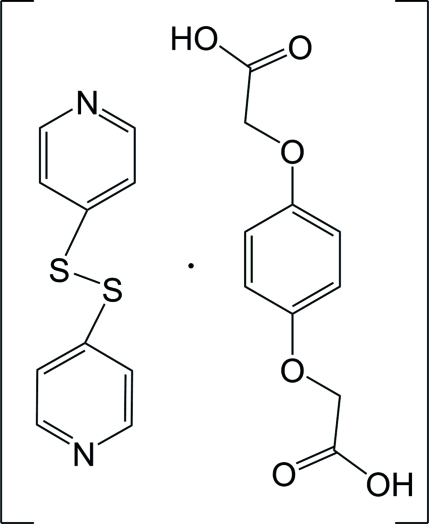


## Experimental

### 

#### Crystal data

C_10_H_8_N_2_S_2_·C_10_H_10_O_6_
*M* *_r_* = 446.50
Monoclinic, 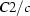

*a* = 14.331 (1) Å
*b* = 5.057 (1) Å
*c* = 28.003 (3) Å
β = 90.200 (5)°
*V* = 2029.4 (5) Å^3^
*Z* = 4
Mo *K*α radiation
μ = 0.30 mm^−1^
*T* = 296 K
0.31 × 0.21 × 0.09 mm

#### Data collection

Bruker APEXII CCD area-detector diffractometer
Absorption correction: multi-scan (*SADABS*; Bruker, 2001 ▶) *T*_min_ = 0.912, *T*_max_ = 0.974
4893 measured reflections
1761 independent reflections
1450 reflections with *I* > 2σ(*I*)
*R*_int_ = 0.026

#### Refinement

*R*[*F* ^2^ > 2σ(*F* ^2^)] = 0.043
*wR*(*F* ^2^) = 0.114
*S* = 1.05
1761 reflections
137 parameters
H-atom parameters constrained
Δρ_max_ = 0.62 e Å^−3^
Δρ_min_ = −0.29 e Å^−3^

### 

Data collection: *APEX2* (Bruker, 2007 ▶); cell refinement: *SAINT* (Bruker, 2007 ▶); data reduction: *SAINT*; program(s) used to solve structure: *SIR97* (Altomare *et al.*, 1999 ▶); program(s) used to refine structure: *SHELXL97* (Sheldrick, 2008 ▶); molecular graphics: *SHELXTL* (Sheldrick, 2008 ▶); software used to prepare material for publication: *WinGX* (Farrugia, 1999 ▶).

## Supplementary Material

Crystal structure: contains datablock(s) global, I. DOI: 10.1107/S1600536811025694/zs2125sup1.cif

Structure factors: contains datablock(s) I. DOI: 10.1107/S1600536811025694/zs2125Isup2.hkl

Additional supplementary materials:  crystallographic information; 3D view; checkCIF report

## Figures and Tables

**Table 1:** Hydrogen-bond geometry (Å, °) *Cg*1 is the centroid of the C8–C10/C8′–C10′ ring.

*D*—H⋯*A*	*D*—H	H⋯*A*	*D*⋯*A*	*D*—H⋯*A*
O3—H3⋯N1^i^	0.82	1.81	2.629 (3)	174
C7—H7*B*⋯*Cg*1^ii^	0.97	2.76	3.528 (2)	136

Symmetry codes: (i) 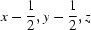; (ii) 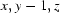.

**Fig. 1.**
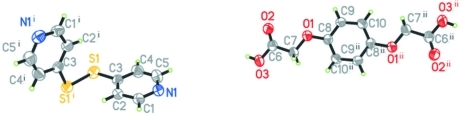
Atom numbering scheme and anisotropic displacement ellipsoid plot of (I) at the 50% probability level. H atoms are represented by circles of arbitrary size. Symmetry codes: (i) -x + 2, y, -z + 1/2; (ii) -x, -y + 2, -z.

**Fig. 2.**
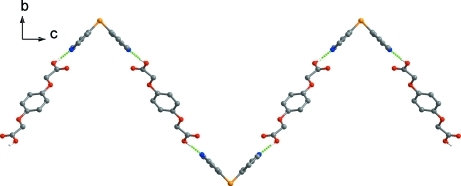
The one-dimensional zigzag chain structure of the title compound. Non-associative H atoms are omitted and hydrogen bonds are shown as dashed lines.

**Fig. 3.**
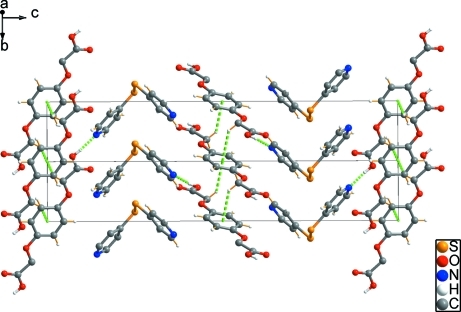
The packing diagram of the title compound showing C—H···π interactions.

## supplementary crystallographic information

### Comment

Bis(4-pyridyl)disulfide (bpds) is often used as a linker in the construction of coordination polymers because of its flexibility (Kondo *et al.*, 2000; Zhu *et al.*, 2010). The attempt at synthesizing a Cd^II^ coordination polymer using bis(4-pyridyl)disulfide and hydroquinone-*O,O*'-diacetic acid (H_2_qda) as ligands gave instead the 1:1 title co-crystal C_10_H_8_N_2_S_2_ . C_10_H_10_O_6_, and the crystal structure is reported here.

In the title compound, the asymmetric unit comprises two half molecules, the bis(4-pyridyl)disulfide having twofold rotational symmetry and the hydroquinone-*O,O'*-diacetic acid having crystallographic inversion symmetry (Fig. 1). In the disulfide molecule, the dihedral angle between the two pyridine rings is 93.2 (1)° while the carboxylic acid groups of the substituted quinone molecule lie essentially in the plane of the benzene ring [dihedral angle, 5.3 (1)°]. In the crystal, the two components are linked *via* intermolecular O—H···N hydrogen bonds into one-dimensional zigzag chains which extend along *c* (Fig. 2) and are inter-linked through C—H···π associations (Table 1, Fig. 3).

### Experimental

A mixture of hydroquinone-*O,O*'-diacetic acid (H_2_qda) (0.023 g, 0.1 mmol), bis(4-pyridyl)disulfide (bpds) (0.022 g, 0.1 mmol) and Cd(NO_3_)_2_ . 4H_2_O (0.038 g, 0.1 mmol) in H_2_O (7.0 ml) was placed in a 16 ml Teflon-lined stainless steel vessel and heated to 160 °C for 48 h, then cooled to room temperature at a rate of -5 °C/h. The solution was filtered and the colorless filtrate was allowed to stand at room temperature. Slow evaporation for about one week afforded colorless block crystals.

### Refinement

All H atoms bonded to C atoms were added according to theoretical models, assigned isotropic displacement parameters and allowed to ride on their respective parent atoms [C—H = 0.93–0.97 Å and *U*_iso_(H) = 1.2*U*_eq_(C)]. The carboxylic acid H atom was located from the Fourier map and allowed to ride on the parent O atom in the final cycles of refinement, with the O—H distance being fixed at 0.82 Å with *U*_iso_(H) = 1.5*U*_eq_(O).

### Crystal data

**Table:**

C_10_H_8_N_2_S_2_·C_10_H_10_O_6_	*F*(000) = 928
*M**_r_* = 446.50	*D*_x_ = 1.461 Mg m^−^^3^
Monoclinic, *C*2/*c*	Mo *K*α radiation, λ = 0.71073 Å
Hall symbol: -C 2yc	Cell parameters from 1634 reflections
*a* = 14.331 (1) Å	θ = 2.8–25.2°
*b* = 5.057 (1) Å	µ = 0.30 mm^−^^1^
*c* = 28.003 (3) Å	*T* = 296 K
β = 90.200 (5)°	Block, colorless
*V* = 2029.4 (5) Å^3^	0.31 × 0.21 × 0.09 mm
*Z* = 4	

### Data collection

**Table:**

Bruker APEXII CCD area-detector diffractometer	1761 independent reflections
Radiation source: fine-focus sealed tube	1450 reflections with *I* > 2σ(*I*)
graphite	*R*_int_ = 0.026
φ and ω scans	θ_max_ = 25.0°, θ_min_ = 1.5°
Absorption correction: multi-scan (*SADABS*; Bruker, 2001)	*h* = −10→16
*T*_min_ = 0.912, *T*_max_ = 0.974	*k* = −5→5
4893 measured reflections	*l* = −33→32

### Refinement

**Table:**

Refinement on *F*^2^	Primary atom site location: structure-invariant direct methods
Least-squares matrix: full	Secondary atom site location: difference Fourier map
*R*[*F*^2^ > 2σ(*F*^2^)] = 0.043	Hydrogen site location: inferred from neighbouring sites
*wR*(*F*^2^) = 0.114	H-atom parameters constrained
*S* = 1.05	*w* = 1/[σ^2^(*F*_o_^2^) + (0.0548*P*)^2^ + 1.8236*P*] where *P* = (*F*_o_^2^ + 2*F*_c_^2^)/3
1761 reflections	(Δ/σ)_max_ < 0.001
137 parameters	Δρ_max_ = 0.62 e Å^−^^3^
0 restraints	Δρ_min_ = −0.29 e Å^−^^3^

### Special details

**Table:**

Geometry. All e.s.d.'s (except the e.s.d. in the dihedral angle between two l.s. planes) are estimated using the full covariance matrix. The cell e.s.d.'s are taken into account individually in the estimation of e.s.d.'s in distances, angles and torsion angles; correlations between e.s.d.'s in cell parameters are only used when they are defined by crystal symmetry. An approximate (isotropic) treatment of cell e.s.d.'s is used for estimating e.s.d.'s involving l.s. planes.
Refinement. Refinement of *F*^2^ against ALL reflections. The weighted *R*-factor *wR* and goodness of fit *S* are based on *F*^2^, conventional *R*-factors *R* are based on *F*, with *F* set to zero for negative *F*^2^. The threshold expression of *F*^2^ > σ(*F*^2^) is used only for calculating *R*-factors(gt) *etc*. and is not relevant to the choice of reflections for refinement. *R*-factors based on *F*^2^ are statistically about twice as large as those based on *F*, and *R*- factors based on ALL data will be even larger.

### Fractional atomic coordinates and isotropic or equivalent isotropic displacement parameters (Å^2^)

**Table:**

	*x*	*y*	*z*	*U*_iso_*/*U*_eq_	
S1	0.92972 (4)	−0.22476 (13)	0.24909 (2)	0.0514 (2)	
O1	0.07968 (11)	0.6344 (3)	0.06109 (5)	0.0485 (4)	
O2	0.15067 (12)	0.2818 (4)	0.12321 (6)	0.0596 (5)	
N1	0.83733 (14)	0.3882 (5)	0.14055 (7)	0.0543 (6)	
O3	0.26328 (12)	0.1757 (4)	0.07197 (7)	0.0683 (6)	
H3	0.2843	0.0923	0.0947	0.102*	
C8	0.04234 (15)	0.8125 (4)	0.02905 (8)	0.0389 (5)	
C9	−0.02803 (15)	0.9711 (5)	0.04649 (8)	0.0440 (6)	
H9	−0.0471	0.9519	0.0780	0.053*	
C7	0.15542 (15)	0.4802 (5)	0.04525 (8)	0.0465 (6)	
H7A	0.2061	0.5945	0.0353	0.056*	
H7B	0.1364	0.3737	0.0181	0.056*	
C3	0.89942 (16)	0.0216 (4)	0.20664 (8)	0.0425 (5)	
C6	0.18774 (15)	0.3037 (5)	0.08524 (9)	0.0462 (6)	
C1	0.92798 (17)	0.3451 (5)	0.14718 (8)	0.0517 (6)	
H1	0.9703	0.4408	0.1289	0.062*	
C2	0.96216 (16)	0.1654 (5)	0.17981 (8)	0.0476 (6)	
H2	1.0261	0.1414	0.1837	0.057*	
C10	−0.07061 (15)	1.1580 (5)	0.01785 (8)	0.0443 (6)	
H10	−0.1181	1.2636	0.0300	0.053*	
C4	0.80519 (17)	0.0660 (6)	0.19985 (10)	0.0584 (7)	
H4	0.7612	−0.0283	0.2173	0.070*	
C5	0.77771 (19)	0.2495 (6)	0.16724 (11)	0.0639 (8)	
H5	0.7142	0.2800	0.1633	0.077*	

### Atomic displacement parameters (Å^2^)

**Table:**

	*U*^11^	*U*^22^	*U*^33^	*U*^12^	*U*^13^	*U*^23^
S1	0.0554 (4)	0.0495 (4)	0.0492 (4)	−0.0077 (3)	−0.0039 (3)	0.0034 (3)
O1	0.0518 (10)	0.0482 (10)	0.0455 (9)	0.0114 (8)	0.0044 (7)	0.0065 (8)
O2	0.0509 (10)	0.0769 (13)	0.0511 (10)	0.0127 (9)	0.0051 (8)	0.0143 (9)
N1	0.0542 (13)	0.0617 (14)	0.0470 (11)	0.0169 (11)	−0.0012 (9)	0.0002 (10)
O3	0.0555 (11)	0.0901 (15)	0.0593 (11)	0.0314 (10)	0.0075 (9)	0.0199 (10)
C8	0.0398 (12)	0.0344 (12)	0.0426 (12)	−0.0008 (9)	−0.0022 (9)	0.0013 (9)
C9	0.0490 (13)	0.0446 (14)	0.0384 (12)	0.0034 (11)	0.0044 (10)	0.0003 (10)
C7	0.0410 (12)	0.0475 (15)	0.0508 (13)	0.0035 (10)	0.0000 (10)	0.0048 (11)
C3	0.0492 (13)	0.0423 (13)	0.0361 (11)	0.0021 (10)	−0.0012 (9)	−0.0069 (10)
C6	0.0397 (13)	0.0484 (14)	0.0504 (14)	−0.0009 (11)	−0.0047 (11)	0.0023 (11)
C1	0.0531 (15)	0.0570 (16)	0.0451 (13)	0.0090 (12)	0.0057 (11)	0.0037 (12)
C2	0.0424 (13)	0.0553 (15)	0.0452 (13)	0.0083 (11)	0.0005 (10)	0.0001 (11)
C10	0.0426 (12)	0.0420 (13)	0.0481 (13)	0.0054 (10)	0.0044 (10)	−0.0011 (10)
C4	0.0454 (14)	0.0656 (18)	0.0643 (16)	−0.0013 (13)	0.0037 (12)	0.0045 (14)
C5	0.0468 (15)	0.075 (2)	0.0701 (18)	0.0106 (14)	−0.0039 (13)	0.0013 (16)

### Geometric parameters (Å, °)

**Table:**

S1—C3	1.775 (2)	C7—C6	1.504 (3)
S1—S1^i^	2.0150 (14)	C7—H7A	0.9700
O1—C8	1.378 (3)	C7—H7B	0.9700
O1—C7	1.409 (3)	C3—C2	1.381 (3)
O2—C6	1.195 (3)	C3—C4	1.381 (3)
N1—C1	1.330 (3)	C1—C2	1.378 (3)
N1—C5	1.336 (4)	C1—H1	0.9300
O3—C6	1.316 (3)	C2—H2	0.9300
O3—H3	0.8200	C10—C8^ii^	1.384 (3)
C8—C9	1.379 (3)	C10—H10	0.9300
C8—C10^ii^	1.384 (3)	C4—C5	1.359 (4)
C9—C10	1.380 (3)	C4—H4	0.9300
C9—H9	0.9300	C5—H5	0.9300
			
C3—S1—S1^i^	105.02 (8)	O2—C6—O3	125.0 (2)
C8—O1—C7	117.02 (17)	O2—C6—C7	125.5 (2)
C1—N1—C5	117.5 (2)	O3—C6—C7	109.5 (2)
C6—O3—H3	109.5	N1—C1—C2	123.1 (2)
C9—C8—O1	115.61 (19)	N1—C1—H1	118.5
C9—C8—C10^ii^	119.4 (2)	C2—C1—H1	118.5
O1—C8—C10^ii^	125.0 (2)	C1—C2—C3	118.5 (2)
C8—C9—C10	121.0 (2)	C1—C2—H2	120.7
C8—C9—H9	119.5	C3—C2—H2	120.7
C10—C9—H9	119.5	C9—C10—C8^ii^	119.7 (2)
O1—C7—C6	109.23 (19)	C9—C10—H10	120.2
O1—C7—H7A	109.8	C8^ii^—C10—H10	120.2
C6—C7—H7A	109.8	C5—C4—C3	119.0 (2)
O1—C7—H7B	109.8	C5—C4—H4	120.5
C6—C7—H7B	109.8	C3—C4—H4	120.5
H7A—C7—H7B	108.3	N1—C5—C4	123.4 (2)
C2—C3—C4	118.5 (2)	N1—C5—H5	118.3
C2—C3—S1	125.16 (18)	C4—C5—H5	118.3
C4—C3—S1	116.30 (18)		
			
C7—O1—C8—C9	−176.4 (2)	C5—N1—C1—C2	0.3 (4)
C7—O1—C8—C10^ii^	3.2 (3)	N1—C1—C2—C3	0.5 (4)
O1—C8—C9—C10	179.7 (2)	C4—C3—C2—C1	−0.5 (3)
C10^ii^—C8—C9—C10	0.1 (4)	S1—C3—C2—C1	178.12 (18)
C8—O1—C7—C6	178.70 (18)	C8—C9—C10—C8^ii^	−0.1 (4)
S1^i^—S1—C3—C2	3.2 (2)	C2—C3—C4—C5	−0.2 (4)
S1^i^—S1—C3—C4	−178.13 (17)	S1—C3—C4—C5	−179.0 (2)
O1—C7—C6—O2	5.5 (3)	C1—N1—C5—C4	−1.1 (4)
O1—C7—C6—O3	−174.7 (2)	C3—C4—C5—N1	1.1 (4)

Symmetry codes: (i) −*x*+2, *y*, −*z*+1/2; (ii) −*x*, −*y*+2, −*z*.

### Hydrogen-bond geometry (Å, °)

**Table:**

Cg1 is the centroid of the C8–C10/C8'–C10' ring.

**Table:**

*D*—H···*A*	*D*—H	H···*A*	*D*···*A*	*D*—H···*A*
O3—H3···N1^iii^	0.82	1.81	2.629 (3)	174
C7—H7B···Cg1^iv^	0.97	2.76	3.528 (2)	136

Symmetry codes: (iii) *x*−1/2, *y*−1/2, *z*; (iv) *x*, *y*−1, *z*.

## References

[bb1] Altomare, A., Burla, M. C., Camalli, M., Cascarano, G. L., Giacovazzo, C., Guagliardi, A., Moliterni, A. G. G., Polidori, G. & Spagna, R. (1999). *J. Appl. Cryst.* **32**, 115–119.

[bb2] Bruker (2001). *SADABS* Bruker AXS Inc., Madison, Wisconsin, USA.

[bb3] Bruker (2007). *APEX2* and *SAINT* Bruker AXS Inc., Madison, Wisconsin, USA.

[bb4] Farrugia, L. J. (1999). *J. Appl. Cryst.* **32**, 837–838.

[bb5] Kondo, M., Shimamura, M., Noro, S., Kimura, Y., Uemura, K. & Kitagawa, S. (2000). *J. Solid State Chem.* **152**, 113–119.

[bb6] Sheldrick, G. M. (2008). *Acta Cryst.* A**64**, 112–122. 10.1107/S0108767307043930 18156677

[bb7] Zhu, H.-L., Zhang, J. & Lin, J.-L. (2010). *Acta Cryst.* E**66**, m185. 10.1107/S1600536810001716 PMC2979707 21579653

